# Neuromuscular Age-Related Adjustment of Gait When Moving Upwards and Downwards

**DOI:** 10.3389/fnhum.2021.749366

**Published:** 2021-10-21

**Authors:** Arthur H. Dewolf, Francesca Sylos-Labini, Germana Cappellini, Dmitry Zhvansky, Patrick A. Willems, Yury Ivanenko, Francesco Lacquaniti

**Affiliations:** ^1^Department of Systems Medicine and Center of Space Biomedicine, University of Rome Tor Vergata, Rome, Italy; ^2^Laboratory of Neuromotor Physiology, IRCCS Santa Lucia Foundation, Rome, Italy; ^3^Department of Pediatric Neurorehabilitation, IRCCS Santa Lucia Foundation, Rome, Italy; ^4^Laboratory of Neurobiology of Motor Control, Institute for Information Transmission Problems, Moscow, Russia; ^5^Laboratoire de Physiologie et Biomecanique de la Locomotion, Université catholique de Louvain, Ottignies-Louvain-la-Neuve, Belgium

**Keywords:** aging, neuromechanics of gait, muscle activity analysis, coordination, spinal motoneuronal output, stair and slope

## Abstract

Locomotor movements are accommodated to various surface conditions by means of specific locomotor adjustments. This study examined underlying age-related differences in neuromuscular control during level walking and on a positive or negative slope, and during stepping upstairs and downstairs. Ten elderly and eight young adults walked on a treadmill at two different speeds and at three different inclinations (0°, +6°, and −6°). They were also asked to ascend and descend stairs at self-selected speeds. Full body kinematics and surface electromyography of 12 lower-limb muscles were recorded. We compared the intersegmental coordination, muscle activity, and corresponding modifications of spinal motoneuronal output in young and older adults. Despite great similarity between the neuromuscular control of young and older adults, our findings highlight subtle age-related differences in all conditions, potentially reflecting systematic age-related adjustments of the neuromuscular control of locomotion across various support surfaces. The main distinctive feature of walking in older adults is a significantly wider and earlier activation of muscles innervated by the sacral segments. These changes in neuromuscular control are reflected in a reduction or lack of propulsion observed at the end of stance in older adults at different slopes, with the result of a delay in the timing of redirection of the centre-of-mass velocity and of an unanticipated step-to-step transition strategy.

## Introduction

Aging causes various motor (McGibbon, 2003; Perry et al., 2007) and sensory deficits (Skinner et al., 1984; Seidler et al., 2010). Since locomotor activities require a complex interaction of musculoskeletal and neural systems, age-related changes in the gait features can be observed (Winter et al., 1990; Samson et al., 2001; Laufer, 2005; Dewolf et al., 2019b). Indeed, numerous studies have described neuromuscular adjustments of gait to age-related physiological changes (e.g., Monaco et al., 2009; Hortobágyi et al., 2016; Gueugnon et al., 2019). In particular, compared to young, older adults’ gait is characterized by shorter step length, longer relative duration of the stance phase, wider burst of muscle activity (Monaco et al., 2010; Santuz et al., 2020), and a distal to proximal shift of joint moments (DeVita and Hortobagyi, 2000; Silder et al., 2008; Anderson and Madigan, 2014; Franz and Kram, 2014). In turn, this modification of distal joint moment has an impact on gait kinetics and kinematics (Noble and Prentice, 2008; Bleyenheuft and Detrembleur, 2012; Hafer and Boyer, 2018; Dewolf et al., 2019b; Gueugnon et al., 2019).

In healthy young adults, locomotor movements can be accommodated to various situations, such as walking slopes (Hong et al., 2014a, b; Dewolf et al., 2017) or stepping on stairs (McFadyen and Winter, 1988; Silverman et al., 2014), by means of appropriate changes in the intersegmental coordination and muscle activations (Dewolf et al., 2018, 2020b). During walking uphill and upstairs, the height of the center of mass of the body (COM) must be increased each step whereas it must be decreased during walking downhill and downstairs. These adjustments result in changes of positive and negative COM muscular power production/absorption (Dewolf et al., 2019a) and a redistribution of joint moments (Alexander et al., 2017; Montgomery and Grabowski, 2018). In turn, changes in the mechanical demand involve modifications in the neuromuscular control during slope walking (Janshen et al., 2017; Rozumalski et al., 2017; Saito et al., 2018). In particular, because biomechanical mechanisms of locomotion are tightly correlated with specific motor pool activations in the spinal cord (Cappellini et al., 2010), slope walking is associated with a different involvement of the lumbar and sacral motor pools (Dewolf et al., 2019a).

In older adults, altered coordination patterns among the elevation angles of the lower limb segments (Noble and Prentice, 2008; Bleyenheuft and Detrembleur, 2012; Dewolf et al., 2019b; Gueugnon et al., 2019), wider bursts of muscle activity (Santuz et al., 2020; Dewolf et al., 2021) and differential organization of the spinal output (Monaco et al., 2010) have been previously documented for level walking. When stepping on stairs, the strategy adopted by the older adults differs from that of young adults, since older apply knee and ankle moments differently from those of young adults (Reeves et al., 2009). Also, the propulsive moment generated by the trailing leg is reduced in older adults, which impacts the step-to-step transition (Meurisse et al., 2019), especially on positive slopes (Franz and Kram, 2014). On the contrary, the reduced contribution of ankle moment during downhill and downstairs stepping (Lay et al., 2006; Montgomery and Grabowski, 2018) may lessen the age-related modification of gait. The purpose of the present study was therefore to provide quantitative comparison of the output of spinal pattern generators between healthy young and older adults when they modify their power requirements to move up and down. In particular, we investigated the intersegmental coordination, muscle activity and the estimated output of motoneuron (MN) pools located at different spinal level during level and slope walking, as well as during upstairs and downstairs stepping in young and older adults. We expect that some neuromuscular age-related adjustments of gait would be present in all conditions, reflecting specific features of locomotion in older adults. We also tested the hypothesis that greater age-related modifications should be observed during tasks with a greater demand for ankle power generation, and *vice-versa*.

## Materials and Methods

### Participants and Experimental Procedure

Eight young healthy adults (4 males – 4 females; age: 28.4 ± 5.2 years old, mass: 76.4 ± 10.1 kg, height: 1.76 ± 0.05 m, means ± SD) and 10 elderly healthy adults (9 males – 1 female; age: 73.5 ± 4.5 years old, mass: 81.5 ± 5.9 kg, height: 1.76 ± 0.05 m, mean ± SD) participated in the study. Mass and height were not significantly different between young and elderly adults (mass: *t* = 0.5, *p* = 0.605; height: *t* = 1.8, *p* = 0.086). No subject had a recent history of falling; they were able to walk without assistance and did not complain about any musculoskeletal disorders. None of the participants had hearing loss or other pathology that could affect navigation performance and mental spatial representation. All subjects gave their informed written consent. Experiments were performed according to the Declaration of Helsinki and were approved by the local ethics committee of IRCCS Fondazione Santa Lucia (protocol n CE/PROG749).

**FIGURE 1:**
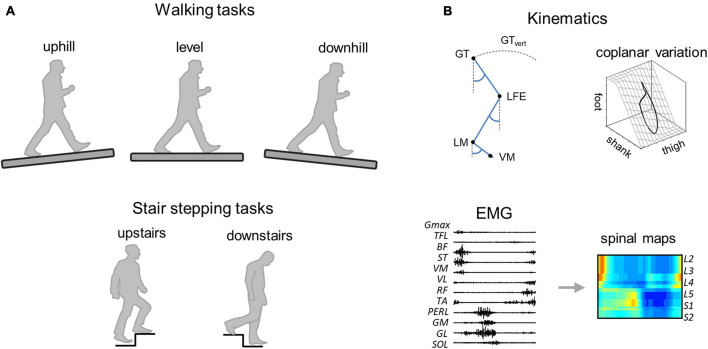
Methodological figure. **(A)** Walking (uphill, level, and downhill) and stair stepping (upstairs and downstairs) tasks. **(B)** Data analysis. GT, greater trochanter; LFE, lateral femoral epicondyle; LM, lateral malleolus; VM, fifth metatarso-phalangeal joint, and GT_vert_, vertical displacement of GT.

Subjects were asked to walk wearing their own walking shoes on a treadmill (En-Mill; 1.1 × 0.5 m) at two different imposed speeds [0.56 (2) and 1.11 (4) m s^–1^(km h−1)] and on three different slopes (0° and ±6°) (Figure 1). One elderly adult was unable to walk uphill and downhill at 1.11 m s^–1^ and one young adult was not tested during stair stepping. Between 10 and 15 strides per trial were recorded; a total of 1,540 strides were analyzed. After a short break, the subjects were asked to perform a series of stepping tasks on stairs. The staircase was composed of two wooden steps with a rise height of 13 cm each and depth of 24.5 cm. The subjects performed five repetitions of step ascent followed by step descent, after remaining stationary for a moment at the top of the staircase. Every ascent/descent was performed with the right leg first and ended in double support on top/bottom of the steps. Each subject performed the tasks at a freely chosen speed.

### Data Recordings

Bilateral, full-body three-dimensional (3D) kinematics were recorded at 200 Hz by means of a Vicon-612 system (Oxford, United Kingdom) with nine cameras placed around the treadmill or the staircase. Twelve reflective markers were attached to the skin of the subjects overlying the following bilateral landmarks: gleno-humeral joint, lateral epicondyle of the elbow, ulnar process of the wrist, greater trochanter, lateral femoral epicondyle, and lateral malleolus. In addition, four markers were placed on each shoe in approximate correspondence with the heel and fifth metatarso-phalangeal joint. The electromyogram (EMG) data were recorded at 2,000 Hz by means of a Delsys Trigno Wireless System (Boston, MA, United States). The following 12 muscles were recorded on the right side of the body: *gluteus maximus* (Gmax), *tensor fasciae latae* (TFL), *vastus medialis* (VM), *vastus lateralis* (VL), *rectus femoris* (RF), long head of the *biceps femoris*, (BF), *semitendinosus* (ST), *tibialis anterior* (TA), *medial gastrocnemius* (GM), *lateral gastrocnemius* (LG), *soleus* (SOL), and *peroneus longus* (PERL). EMG electrodes were placed based on recommendation of SENIAM,^1^ the European project on surface EMG. To ensure placement of EMG electrodes, muscle bellies were located by means of palpation and the electrodes were oriented along the main direction of the fibers (Kendall et al., 2005). In certain conditions, some electrodes became partially detached and were removed from the analysis on a subject-specific basis. Out of a total of 1,476 EMG recordings, we lost 8% of them. Kinematic and EMG recordings were synchronized on-line.

### Kinematic Data Analysis

During walking, the stride was defined as the period between two contacts of the right foot with the ground. During stepping on stairs, the stride was defined as the period between two lift-offs of the right foot (in order to have one complete cycle). Foot-contact/lift-off were estimated according to the local minima of the vertical displacement of the heel/fifth metatarso-phalangeal joint markers, respectively (Ivanenko et al., 2007).

From the marker locations, the orientation of the thigh, shank, foot, and trunk relative to the vertical axis (elevation angle) were computed as described in Borghese et al. (1996). For each subject, the different strides of each trial were normalized by interpolating individual gait cycles over 200 points (i.e., each point corresponding to 0.5% of the stride). To analyze the relative phase of the time-course of the elevation angles during a stride, the phase lags between two adjacent limb-segments were computed by means of cross-correlation function. The trajectory of the COM was estimated by the trajectory of a point located at mid-distance between left and right greater trochanters. The trajectory of this point has been shown to be similar to the trajectory of the COM, estimated from the ground reaction forces (Dewolf et al., 2018). From the coordinates of this point, we estimated the displacement and velocity of the COM.

In order to determine the covariance matrix of the segment elevation angles, a principal component analysis was applied. The eigenvalues and eigenvectors *u*_i_ were computed by factoring the covariance matrix from the set of original signals by using a singular value decomposition algorithm. The first two eigenvectors (*u*_1_ and *u*_2_) lie on the best-fitting plane of angular covariation, and the data projected onto the corresponding axes correspond to the first (PC_1_) and second (PC_2_) principal components. The planarity was evaluated for each condition by calculating the percentage of variance that was explained by *u*_1_ (PV_1_), *u*_2_ (PV_2_), and *u*_3_ (PV_3_). If the data lie perfectly on a plane, PV_3_ would be 0%. By definition, the third eigenvector *u*_3_ is orthogonal to the plane defined by *u*_1_ and *u*_2_. The parameter *u*_3__t_ corresponds to the direction cosine with the positive semi-axis of the thigh, and provides a measure of the orientation of the plane (Bianchi et al., 1998).

### Electromyogram Data Analysis

The collected raw EMG signals were high-pass filtered (30 Hz), then rectified and low-pass filtered with a zero-lag 4th-order Butterworth filter (10 Hz). A custom-made automatic search for artifacts was also implemented, by comparing the EMG envelope of each gait cycle with the average envelope. The time scale was normalized by interpolating individual gait cycles over 200 points (i.e., every 0.5%). For each condition and for each EMG waveform, the full width at half maximum (FWHM) was calculated as the period during which the EMG activity exceeded half of its maximum (Martino et al., 2014; Dewolf et al., 2020b; Santuz et al., 2020). The center of activity (CoA) of each EMG waveform was also calculated as the angle of the vector that points to the center of mass of the circular distribution (Martino et al., 2014).

The EMG activities were then mapped onto the estimated rostro-caudal location of the MN pools in the human spinal cord from the L2 to S2 segments. This reconstruction is based on the approximate location of MN pools innervating different muscles in the human spinal cord based on published charts of segmental localization (Kendall et al., 2005), as in Ivanenko et al. (2006, 2013). In general, each muscle is innervated by several spinal segments (Table 1). To account for size differences in MN pools at each spinal level, this fractional activity value was then multiplied by the estimated segment-specific number of MNs (MN_j_), based on Tomlinson and Irving (1977).

**TABLE 1:** Innervation of the lower limb muscles.

	**Gmax**	**TFL**	**BF**	**ST**	**VM**	**VL**	**RF**	**TA**	**PERL**	**GM**	**GL**	**SOL**
L2					X	X	X					
L3					X	X	X					
L4		X		x	X	X	X	X	x			
L5	X	X	x	X				X	X			x
S1	X	X	X	X				X	X	X	X	X
S2	X		X	X						X	X	X

*Reference segmental charts for lower limb muscles from Kendall et al. (2005), obtained by combining the anatomical and clinical data from six different sources. A capital X denotes localization agreed on by five or more sources, a small x denotes agreement of three to four sources.*

To compute the total motor output in each condition, we summed the motor output patterns of each spinal segment over the gait cycle. The minimal activation and the range (maximum-minimum) were then computed. The relative activation of the lumbar and sacral segments in each condition were computed as the average motor output patterns in the upper part of the lumbar segments (sum of the activity from L2 to L4) and the sacral segments (sum of activity from S1 to S2). Note that to reduce overlaps due to maps smoothing, the spinal segment L5 was not taken into account (Dewolf et al., 2019a, 2020a). The FWHM and the timing of the maximal activation were then calculated for both lumbar and sacral segments.

### Statistics

For the walking tasks, the statistical analysis was designed to assess the effect of speed of progression, slope, age group (young vs. older adults), and the interaction between these factors. For the stair tasks, the statistical analysis was designed to assess the effect of step direction (ascent vs. descent), age group, and the interaction between these factors. A linear mixed model was applied. The normality of the residuals was tested by means of the Kolmogorov–Smirnov test. Normality was not assumed for five variables (range of motion of the trunk elevation angle and of the thigh elevation angle during walking, shank-foot phase lag, the FWHM of Gmax and the CoA of RF). In those cases, a log transform was applied, and the normality of the residuals was then assumed. The effect size, measure by the eta square (*η*_p_^2^), is reported for age group comparisons. Circular statistics (Berens, 2009) were used to characterize the CoA of each muscle and spinal output. The Rayleigh test (Berens, 2009) was used to check whether the samples were distributed uniformly around the gait cycle or had a common mean direction. In all analyses, the significance level was fixed at *p* < 0.05.

## Results

### Gait and Kinematic Parameters

**FIGURE 2:**
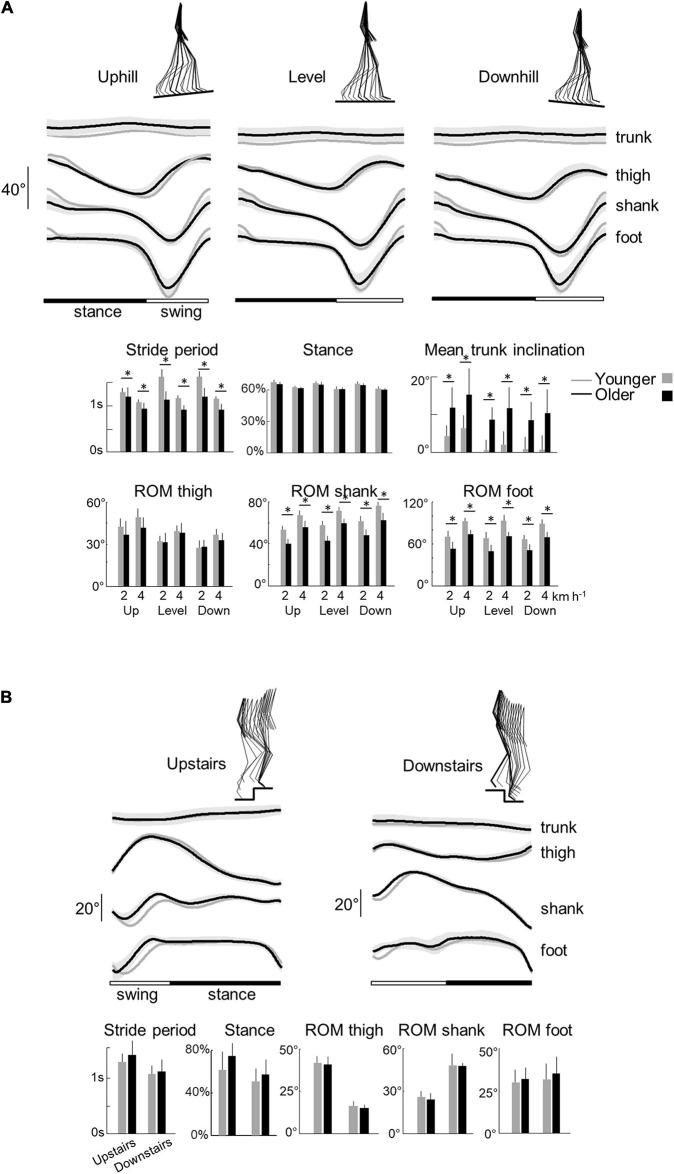
Elevation angles of lower-limb segments and general gait parameters across all conditions in younger and older adults. **(A)** Walking tasks: elevation angles of the trunk, thigh, shank, and foot over a stride. All the curves of each subject walking at a given walking condition were first averaged (mean-curve). The curves presented here are the average of the mean-curves of all the young (gray lines) and older (black lines) adults. The gray zone represents ±1 SD for the older adults. Below, the bar plots present the average stride period, relative stance phase, and mean trunk inclination over one stride, as well as the average range of motion of the thigh, shank, and foot over one stride. Bars are presented for each walking task and at each speed (2 and 4 indicate the speed in km h^–1^). **(B)** Stair stepping tasks: elevation angles of the trunk, thigh, shank, and foot over a stride. Below, the bar plots present the average stride period, relative stance phase, and the average range of motion of the thigh, shank, and foot over one stride. In all panels, gray bars correspond to young adults, whereas black bars correspond to elderly adults. Thin lines represent one standard deviation. The * indicates a significant effect of age (Student *t*-test, *p* < 0.05).

We first report the results of walking tasks. The stride period (and thus stride length) decreased with speed (*F*_1,95_ = 162.8; *p* < 0.001; Figure 2A) but did not change with the slope (*F*_2,95_ = 1.2; *p* = 0.295) in both age groups. At a given speed, older adults stepped at a higher cadence with shorter strides (shorter stride period) than young adults (*F*_1,95_ = 175.1; *p* < 0.001; *η*_p_^2^ = 0.65). However, the effect of speed on the stride period was greater in young adults (stride period: *F*_1,95_ = 4.3; *p* = 0.04).

The relative duration of the stance phase became smaller when speed increased both in young (*F*_1,95_ = 117.4; *p* < 0.001) and older adults (*F*_1,95_ = 6.6.5; *p* = 0.012; *η*_p_^2^ = 0.06). The relative stance period was also slightly affected by the slope (*F*_1,95_ = 3.9; *p* = 0.023). The down-to-up redirection of the COM velocity at step-to-step transition, defined by the time of occurrence of the minimal vertical velocity of the COM [Vv_min_] relative to the beginning of the double contact phase (Franz and Kram, 2013; Meurisse et al., 2019), began later in older than in young adults (*F*_1,95_ = 6.5; *p* = 0.014; *η*_p_^2^ = 0.13) and in downhill than uphill walking (*F*_2,95_ = 27.3; *p* < 0.001). As a result, the transition phase began later in older than in young adults.

When speed increased, the range of motion (ROM) of the thigh, shank, and foot elevation angles increased (thigh: *F*_1,95_ = 29.3; *p* < 0.001; shank: *F*_1,95_ = 230.3; *p* < 0.001; foot: *F*_1,95_ = 201.2; *p* < 0.001) in both groups (Figure 2A). The inclination of the treadmill affected the ROM of thigh and shank angles (thigh: *F*_1,95_ = 28.8.5; *p* < 0.001; *shank: F*_1,95_ = 21.4; *p* < 0.001) but not the ROM of the foot angle (*F*_1,95_ = 1.3; *p* = 0.265). During uphill walking, the thigh ROM increased while the shank ROM decreased as compared to level walking (*p* < 0.001 *Bonferroni post hoc*). The contrary was true for downhill walking (*p* < 0.038 *Bonferroni post hoc*).

Older adults performed smaller amplitude of movement of the different lower-limb segments than young adults (Figure 2A; thigh: *F*_1,95_ = 6.2; *p* = 0.014, *η*_p_^2^ = 0.1; shank: *F*_1,95_ = 176.2, *η*_p_^2^ = 0.65; *p* < 0.001; foot: *F*_1,95_ = 150.3; *p* < 0.001, *η*_p_^2^ = 0.61). In addition, older adults walked with a greater mean forward inclination of the trunk relative to the vertical than young adults (*F*_1,95_ = 89.6; *p* < 0.001; *η*_p_^2^ = 0.49).

**FIGURE 3:**
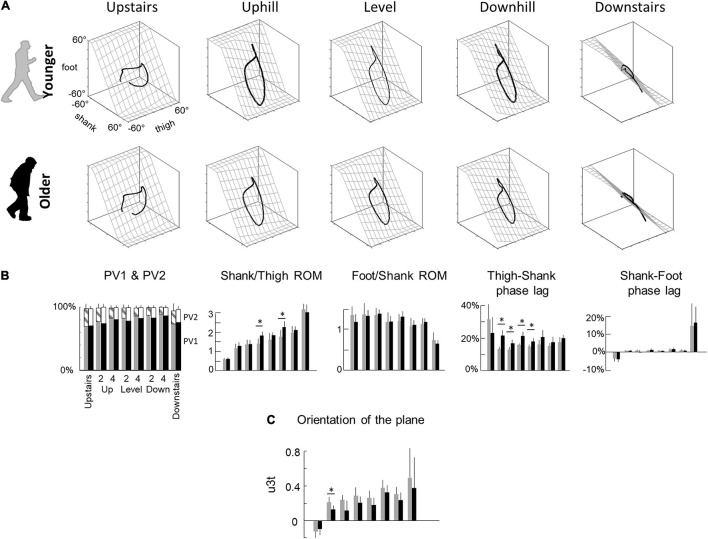
Planar covariation of elevation angles. **(A)** Covariation of the ensemble-average limb-segment elevation angles across young (up) and elderly adults (bottom) in all conditions. Note that when the elevation angles of thigh, shank, and foot are plotted one vs. the other in a x-y-z space, they co-vary along a loop constrained on a plane (x–y). Grids show the best-fitting plane. **(B)** Average percentage of variance accounted for by the first (PV_1_) and second (middle – PV_2_) eigenvector of the principal component analysis, average amplitude ratios between the range of motion of adjacent segments and phase lags between time curves of elevation angles of adjacent segments. Bars are presented for each condition and at each speed (2 and 4 indicate the speed in km h^–1^). **(C)** The direction cosines of the normal to the covariation plane with the positive semi-axis of the thigh angular coordinates (u3t). Other indications as in Figure 2.

With regards to stepping on stairs, older adults were slower than young adults (longer stride period) (*F*_1,32_ = 5.4; *p* = 0.027, *η*_p_^2^ = 0.16; Figure 3B). In both groups, stepping downstairs was performed faster than moving upstairs (*F*_1,35_ = 11.3; *p* = 0.002). The relative duration of the stance phase was longer when stepping upstairs than downstairs (*F*_1,35_ = 6.6; *p* = 0.015) but it did not differ significantly between young and older adults (*F*_1,35_ = 0.1; *p* = 0.739).

Similar to walking on slopes, upstairs stepping involved an increase of the thigh ROM (*F*_1,35_ = 374.7; *p* < 0.001) and a decrease of the shank ROM (*F*_1,35_ = 156.1; *p* < 0.001), as compared to downstairs stepping. No significant difference was found in foot ROM (*F*_1,35_ = 0.6; *p* = 0.418). In addition, we found no significant difference on other kinematic parameters between older and young adults (mean trunk inclination, thigh ROM, shank ROM, and foot ROM: all *p* > 0.407).

### Intersegmental Coordination When Moving Upwards and Downwards

The coordination between thigh, shank and foot elevation angles was evaluated using principal component analysis (Figure 3). Figure 3A illustrates the averaged gait loops plotted in 3D during walking on slopes and stepping on stairs in both age groups. Note significantly smaller loops in elderly adults in the walking conditions (related to the smaller ROM reported in the previous section).

In each condition, PV_1_ + PV_2_ > 97% indicating an excellent fit of a planar regression on the elevation angles of the three lower limb segments (Figure 3B). However, PV_1_ was smaller (and PV_2_ greater) in older than in young adults during walking (PV_1_: *F*_1,95_ = 19.7; *p* < 0.001; *η*_p_^2^ = 0.17; PV2: *F*_1,95_ = 18.3; *p* < 0.001; *η*_p_^2^ = 0.16), but they were similar during stepping on stairs (PV_1_: *F*_1,35_ = 0.2; *p* = 0.606; PV2: *F*_1,35_ = 0.1; *p* = 0.733). Furthermore, PV_1_ slightly increased (and PV_2_ decreased) with walking speed (PV_1_: *F*_1,95_ = 27.1; *p* < 0.001; PV2: *F*_1,95_ = 27.6; *p* < 0.001) and was greater in downhill walking and downstairs negotiation (PV_1_: *F*_2,95_ = 29.8; *p* < 0.001; PV2: *F*_2,95_ = 29.1; *p* < 0.001). The orientation of the plane, shown by the direction cosine *u_3__*t*_* (Figure 3C), rotated across conditions (with treadmill slope: *F*_1,95_ = 23.9; *p* < 0.001; with stair conditions: *F*_1,35_ = 36.1; *p* < 0.001). The *u_3__*t*_* was smaller in upstairs stepping and uphill walking, whereas it was greater in downhill walking and downstairs stepping. During walking, *u_3__*t*_* was smaller in older than in young adults (*F*_1,95_ = 25.8; *p* < 0.001; *η*_p_^2^ = 0.22), whereas no significant difference was observed during stair negotiation (*F*_1,35_ = 0.2; *p* = 0.619). When all conditions were considered together (both stair and slope conditions), the variation of *u_3__*t*_* across conditions was more important in young than in older adults (young: *F*_6,62_ = 11.6; *p* < 0.001; older: *F*_6,76_ = 9.8; *p* = 0.001).

Both the shape of the loop and the orientation of the plane depend on the amplitude ratio and the time relationship characteristics of the elevation angles of adjacent limb segments (Figure 3B). During walking, the ratio between thigh and shank ROM was smaller in elderly adults (*F*_1,95_ = 21.0; *p* < 0.001; *η*_p_^2^ = 0.18) and greater on negative than on positive slopes (*F*_2,95_ = 62.8; *p* < 0.001). Similarly, the ratio between thigh and shank ROM was smaller when stepping upstairs than downstairs (*F*_1,35_ = 814.9; *p* < 0.001), but no difference between older and young adults was observed (*F*_1,35_ = 0.6; *p* = 0.420). The ratio between shank and foot ROM was affected by conditions in an opposite way: it was greater when stepping upstairs and walking uphill and smaller when stepping downstairs and walking downhill (slope: *F*_2,95_ = 15.2; *p* < 0.001; stairs: *F*_1,35_ = 75.8; *p* < 0.001). In all conditions, no effect of age groups was found (walking: *F*_2,95_ = 0.9; *p* = 0.336; stepping: *F*_1,35_ = 3.6; *p* < 0.067). The phase lag between thigh and shank displacement was significantly affected by age during walking (*F*_1,95_ = 81.4; *p* < 0.001; *η*_p_^2^ = 0.47), but not during stepping on stairs (*F*_1,35_ = 1.3; *p* = 0.257). The phase lag between shank and foot was not significantly affected by age, whatever the conditions (slope: *F*_1,95_ = 0.2; *p* = 0.66; stairs: *F*_1,35_ = 0.1; *p* = 0.792).

### Muscle Activations

**FIGURE 4:**
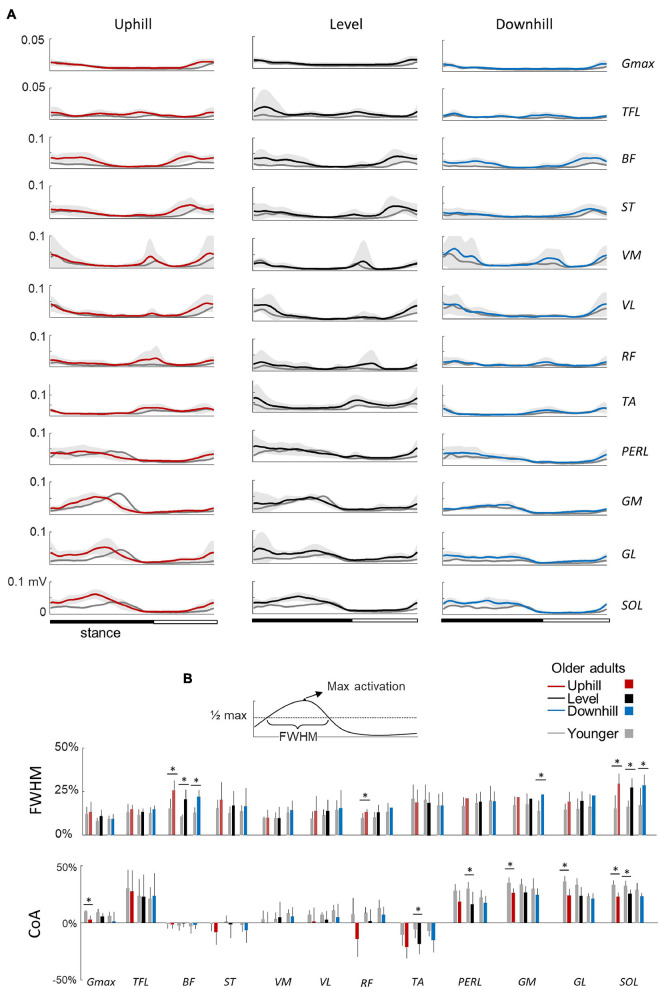
Ensemble-averaged electromyogram (EMG) patterns during uphill, level, and downhill walking in young and older adults. **(A)** Ensemble-averaged EMG patterns over one stride in uphill (red), level (black), and downhill (blue) walking. *Gmax*, gluteus maximus; *TFL*, tensor fascia latae; *VM*, vastus medialis; *VL*, vastus lateralis; *RF*, rectus femoris; *BF*, biceps femoris; *ST*, semitendinous; *TA*, tibialis anterior; *MG*, gastrocnemius medialis; *LG*, lateral gastrocnemius; *SOL*, soleus; *PERL*, peroneus longus. The curves presented here are the average of the mean-curves of all the young (gray lines) and elderly (colored lines) adults. The gray zone represents ±1 SD for the older adults. **(B)** Full Width Half Maximum (FWHM) and center of activity (CoA) of the 12 lower-limb muscles at each walking condition. The bars represent the grand mean of all the young (gray) and the elderly (colored) adults. Thin lines represent one standard deviation. The * indicates a significant effect of age (Student *t*-test, *p* < 0.05).

Figure 4A illustrates ensemble averages of rectified EMG envelopes for all walking conditions in young and older adults. EMGs of young adults are visually consistent with those reported in the literature for level and slope walking (Dewolf et al., 2019a, 2020b). The slopes slightly modified the EMG activity: for example, the FWHM of Gmax, BF, and RF were significantly affected by the slope (*F*_1,95_ = 7.6; *p* = 0.001; *F*_1,95_ = 3.4; *p* = 0.038; *F*_1,95_ = 5.4; *p* = 0.006).

In older adults, EMG data remained qualitatively similar to those of young adults. However, many muscles were characterized by a duration and timing of activation different from those of young adults (Figure 4B). In particular, the CoA of many muscles occurred significantly earlier in the stride (circular statistics: *Gmax*: *F*_1,95_ = 23.4; *p* < 0.001; *ST*: *F*_1,95_ = 7.9; *p* = 0.006; *VL*: *F*_1,95_ = 12.6; *p* < 0.001; *RF*: *F*_1,95_ = 16.1; *p* < 0.001; *TA*: *F*_1,95_ = 75.5; *p* < 0.001; *PERL*: *F*_1,95_ = 69.4; *p* < 0.001; *GM*: *F*_1,95_ = 34.9; *p* < 0.001; *GL*: *F*_1,95_ = 45.8; *p* < 0.001; *SOL*: *F*_1,95_ = 69.9; *p* < 0.001). In addition, older adults displayed longer burst of muscle activations than young adults, as assessed by considering the FWHM in the following muscle groups: hamstrings (*BF*: *F*_1,95_ = 102.2; *p* < 0.001; *η*_p_^2^ = 0.53; *ST*: *F*_1,95_ = 11.5; *p* = 0.001; *η*_p_^2^ = 0.11), knee extensors (VL: *F*_1,95_ = 5.4; *p* = 0.02; *η*_p_^2^ = 0.06; RF: *F*_1,95_ = 17.8; *p* < 0.001; *η*_p_^2^ = 0.17), and ankle extensors (*GM*: *F*_1,95_ = 35.4; *p* < 0.001; *η*_p_^2^ = 0.17; *GL*: *F*_1,95_ = 20.4; *p* < 0.001; *η*_p_^2^ = 0.19; *SOL*: *F*_1,95_ = 68.7; *p* < 0.001; *η*_p_^2^ = 0.44).

**FIGURE 5:**
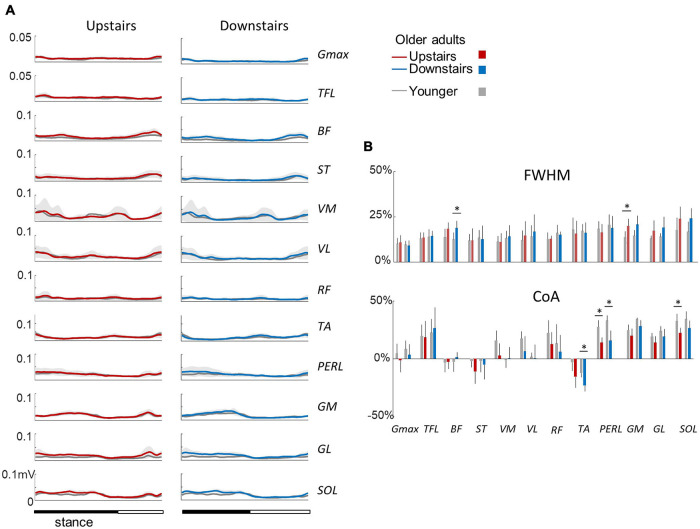
Ensemble-averaged EMG patterns during upstairs and downstairs stepping in young and older adults. **(A)** Ensemble-averaged EMG patterns over one stride in upstairs (red) and downstairs (blue) stepping. **(B)** FWHM and CoA of the 12 lower-limb muscles during stair stepping. Other indications as in Figure 2.

Figure 5A illustrates ensemble averages of rectified EMG envelopes during upstairs and downstairs stepping in young and older adults. Very few differences of EMG duration were observed between upstairs and downstairs: the FWHM of *RF* was significantly greater during downstairs than upstairs stepping (*F*_1,35_ = 4.6; *p* = 0.041; Figure 5B). An effect of age was also observed, but to a lesser extent than for walking. The FWHM of *BF*, *GM*, and *SOL* (*BF*: *F*_1,35_ = 13.2; *p* = 0.001; *η*_p_^2^ = 0.34; *GM*: *F*_1,35_ = 16.8; *p* < 0.001; *η*_p_^2^ = 0.39; *SOL*: *F_1,35_* = 9.0; *p* = 0.006; *η*_p_^2^ = 0.26) and the CoA of distal muscles occurred earlier in older than younger adults (circular statistics: *TA*: *F_1,35_* = 13.4; *p* < 0.001; *PERL*: *F_1,35_* = 36.0; *p* < 0.001; *GM*: *F*_1,35_ = 4.7; *p* = 0.038; *GL*: *F*_1,35_ = 5.2; *p* = 0.03; *SOL*: *F*_1,35_ = 14.8; *p* < 0.001).

### Spinal Motor Output When Moving Upwards and Downwards

**FIGURE 6:**
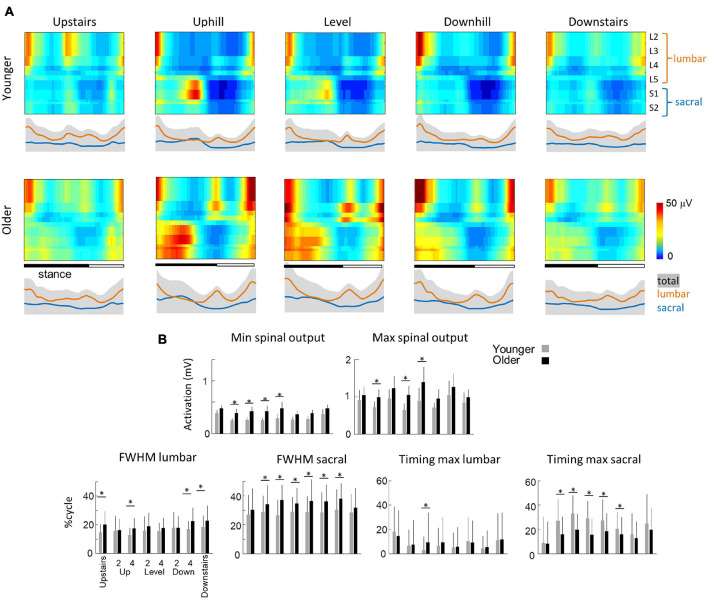
Unilateral spatiotemporal spinal motor outputs computed from ensemble-averaged EMGs across all subjects in all conditions (Table 1). **(A)** Motor output (reported in μV) is plotted as a function of gait cycle in young (top) and older (bottom) adults. The thin green and red lines correspond to the activation of the lumbar and sacral segments, respectively. The gray area is the sum of all segments and reflects the level of activation **(B)** average minimal and maximal level of activation of the spinal motor output, average FWHM, and timing of the maximal activation of the lumbar and sacral segments. Bars are presented for each condition and at each speed (2 and 4 indicate the speed in km h^–1^). Other indications as in Figure 2.

Figure 6A presents the spatio-temporal maps derived from the EMG activities of Figures 4A, 5A. In brief, the recorded patterns of EMG activity were mapped onto the spinal cord in approximate rostrocaudal locations of the MN pools. This approach provides information about pattern output during locomotion in terms of lumbosacral segmental control (from L2 to S2) rather than in terms of individual muscle control. In each condition, both the lumbar and sacral segments presented one major spot of activity (Ivanenko et al., 2006; Cappellini et al., 2010; Dewolf et al., 2019a, 2021). The first burst occurs around foot contact and is mainly localized on the lumbar segment whereas the second burst occurs during the second part of stance mainly and is localized on the sacral segment.

Striking age-related differences were observed in the burst timing and duration of those spots of activity. As compared to young adults, older adults had greater FWHM of the lumbar and sacral MN activation during walking (lumbar: *F*_1,95_ = 46.2; *p* < 0.001; *η*_p_^2^ = 0.23; sacral: *F*_1,95_ = 142.4; *p* < 0.001; *η*_p_^2^ = 0.42) and during stepping on stairs (lumbar: *F*_1,35_ = 33.0; *p* < 0.001; *η*_p_^2^ = 0.20; sacral: *F*_1,35_ = 6.2; *p* = 0.013; *η*_p_^2^ = 0.12). In addition, during walking, the timing of the maximum activation of the sacral segments occurred earlier in old than in young adults (*F*_1,95_ = 148.6; *p* < 0.001; *η*_p_^2^ = 0.46) whereas the timing of maximum activation of the lumbar segments did not change with age (*F*_1,95_ = 1.1; *p* = 0.296). The FWHM of the lumbar and sacral bursts changed as a function of the slope, with a longer burst of lumbar segments during downhill walking (*F*_2,95_ = 16.4; *p* < 0.001) and a longer burst of sacral segment during uphill walking (*F*_2,95_ = 3.1; *p* = 0.046). Also, the timing of the maximal activation of the sacral segments occurred earlier during walking downhill (*F*_2,95_ = 40.2; *p* < 0.001). Note that the effect of age was larger during uphill than during downhill walking (age by slope interaction: *F*_1,95_ = 30.8; *p* < 0.001). During stepping on stair, the FWHM of lumbar and sacral segments were not significantly affected by the direction of progression. The maximum lumbar activation occurred significantly earlier during downstairs stepping (*F*_1,35_ = 11.8; *p* = 0.001) and the maximum sacral activation occurred significantly later during upstairs stepping (*F*_1,35_ = 54.2; *p* < 0.001). No significant effect of age was observed on these two timings (lumbar: *F*_1,35_ = 1.8; *p* = 0.180; sacral: *F*_1,35_ = 2.7; *p* = 0.096).

The minimal level of activity of the lumbar and sacral segments pooled together (total spinal motor output) and the range of activity (maximum-minimum) remained similar across slopes during walking (minimum: *F*_2,95_ = 2.5; *p* = 0.086; range: *F*_2,95_ = 0.2; *p* = 0.821) and during downstairs or upstairs stepping (minimum: *F*_1,35_ = 0.3; *p* = 0.591; range: *F*_1,35_ = 0.4; *p* = 0.528). In each condition, older adults had always a significantly higher minimal level of activity than younger adults (walking: *F*_1,95_ = 82.7; *p* < 0.001; *η*_p_^2^ = 0.48; stepping: *F*_1,35_ = 17.3; *p* < 0.001; *η*_p_^2^ = 0.40). However, a significant age-effect on the range of activation was only observed during walking (*F*_1,95_ = 12.5; *p* < 0.001; *η*_p_^2^ = 0.13) but not during when stepping on stairs (*F*_1,35_ = 0.13; *p* < 0.717). In addition, the co-activation of lumbar and sacral segments was significantly greater in older than in young adults during slope walking (*F*_2,95_ = 3.9; *p* = 0.05; *η*_p_^2^ = 0.06), but it was not significantly different during stepping on stairs (*F*_1,35_ = 0.16; *p* = 0.694).

## Discussion

The purpose of this study was to quantify the effect of aging on the neuromuscular control of gait when moving upwards and downwards. Specifically, we analyzed walking up and down a slope and stepping up and down a stair. As expected, we found common age-related modifications of gait, suggesting specific adjustments of the motor control (Franz, 2016). In all conditions, the activation profiles of ankle extensor and knee flexor muscles were wider and the minimal level of activation of the total spinal output was higher in older than in younger adults. We also found that other age-related differences were present during walking tasks but not when stepping on stairs. As discussed below, the changes may be larger during tasks requiring greater propulsive function of the trailing leg, impacting the step-to-step transitions.

### Widening of Muscle Activation in Older Adults

Our results are consistent with prior results showing specific spatiotemporal feature of gait in older adults during both walking (e.g., Herssens et al., 2018) and stair stepping (Williams and Bird, 1992; Startzell et al., 2000). Thus, older tend to take shorter steps during walking (Figure 2A) and slower steps when stepping on stairs (Figure 2B) than younger adults. In addition, despite the various biomechanical constraints induced by the different situations studied, we observed similar age-related differences on muscle activations, suggesting that gait impairments in older adults cannot depend only on a reduction of force generated by plantar flexor muscles (Franz, 2016). In particular, the activity profiles of the knee flexor and ankle extensor muscles, innervated from the sacral segments, are significantly wider in older adults in all conditions (Figures 4A, 5A), that could be related to a more robust neuromuscular control (i.e., more able to cope with errors) to deal with poorer balance control (Martino et al., 2015; Santuz et al., 2018). The spinal maps of motoneuron activity also clearly illustrates this finding (Figure 6A). Similar widening has already been documented during level walking (Monaco et al., 2010; Santuz et al., 2020; Dewolf et al., 2021).

### Higher Activation of Spinal Motor Pools

Another modification of gait in the elderly, shared across the conditions, is the higher minimal level of the spinal motor output (Figure 6B), which may contribute to the age-related increase in the metabolic cost of locomotion (Mian et al., 2006). There might be other group or individual differences such as level of physical activity, gender, experience with inclined (mountaineering) walking, which could potentially also contribute to the observed differences. However, it is unlikely that these factors alone can account for the present age-related differences since EMG widening and higher activation of motor pools have been previously reported in other population of patients both for males and females and with different level of physical activity (Monaco et al., 2010; Martino et al., 2018; Santuz et al., 2020). Nevertheless, future research investigating the contribution and interaction of different factors would be beneficial to assess gait of older adults. On the one hand, the higher tonic activation could be related to wider muscle activations, also resulting in increased co-activations (Hortobágyi and DeVita, 2000). The most commonly ascribed role to co-activation is to provide mechanical stability by stiffening joints (Hogan, 1984; Martino et al., 2014). On the other hand, the increased minimal level of the spinal motor output may be related to alterations in the peripheral neuromuscular system, including changes in muscle contractile mechanics (Brooks and Faulkner, 1988), reduction in the number and/or size of type II fibers (Lexell, 1995; Aagaard et al., 2007), partial denervation at the neuromuscular junction (Arizono et al., 1984), or muscle atrophy (Rosenberg, 2011). Consequently, the size of motor units and firing rates used to achieve a given force increase with age (Ling et al., 2009).

### Unanticipated Step-to-Step Transition Strategy

During walking, additional age-related differences were present that were not during stepping on stairs. As already documented, older adults adapt their intersegmental coordination and display a different orientation of the covariation plane in all walking conditions (Noble and Prentice, 2008; Bleyenheuft and Detrembleur, 2012; Dewolf et al., 2019b; Gueugnon et al., 2019; Figure 3A), shedding light on age-related modifications of the coordination strategies during walking. In particular, the age-related rotation of the plane occurs mainly along the long axis of the gait loop (Figure 3A), leading to a smaller *u*_3__t_ (Figure 3C). The change in plane orientation is mainly related to a change of the phase shift and range of motion ratio between adjacent lower-limb segments (Borghese et al., 1996; Dewolf et al., 2019b). Most previous studies interpreted the modification of plane orientation in terms of a reduction of ankle plantar-flexion at the end of stance and redistribution of joint moments (Alexander et al., 2017; Montgomery and Grabowski, 2018). Here, we found that the activation of the sacral segments occurred earlier in older as compared to young adults, mainly related to an early activation of ankle plantar-flexor muscles and lack of later activation (Figure 4A).

**FIGURE 7:**
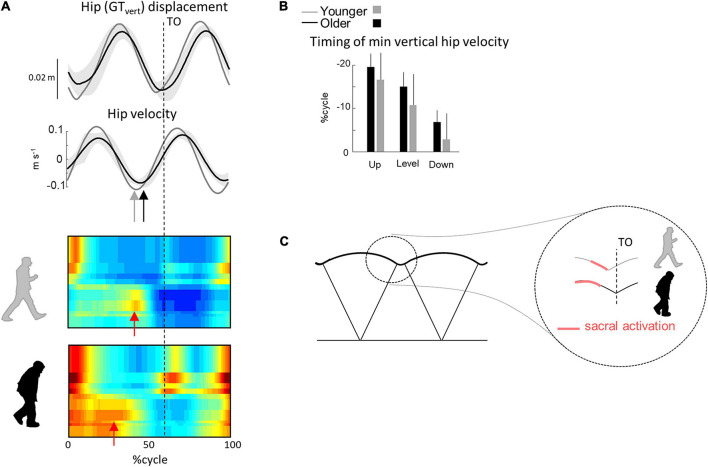
Unilateral spatiotemporal spinal motor outputs computed from ensemble-averaged EMGs across all conditions. **(A)** Motor output (reported in μV) is plotted as a function of gait cycle in young (top) and older (bottom) adults. The thin green and red lines correspond to the activation of the lumbar and sacral segments, respectively. The gray area is the sum of all segments and reflects the level of activation. **(B)** Average minimal and maximal level of activation of the spinal motor output, average FWHM, and timing of the maximal activation of the lumbar and sacral segments. **(C)** Illustration of step-to-step transition. Other indications as in Figure 2.

A comparison of different human gaits and developmental considerations highlight the link existing between COM trajectory and functional spinal cord topography (Cappellini et al., 2010; Dewolf et al., 2020c). The reduction of ankle power generation at the end of stance in turn modifies the step-to-step transition in older (Meurisse et al., 2019). Indeed, due to the lack of late push-off from the trailing leg, the down-to-up redirection of the COM velocity starts later in older than in young adults (Figure 7). This re-direction can be estimated from the time of occurrence of the minimal vertical velocity of the COM relative to the beginning of the double contact phase (Franz and Kram, 2013; Meurisse et al., 2019). This modification of the timing of sacral activation, related to the earlier activation of plantar-flexor muscles, may be adopted by the older adults to use their plantar-flexor muscles over a more favorable portion of the moment–angle relation (Reeves et al., 2009). In addition, we observed here that the age-related difference in sacral activation timing was greater in uphill than in downhill walking. Similarly, it has been observed that the reduced ankle moment in older adults observed during level walking (Monaco et al., 2009; Hortobágyi et al., 2016; Gueugnon et al., 2019) was even more pronounced during uphill walking (Waanders et al., 2020; Krupenevich et al., 2021), indirectly supporting the idea that the change in activity of sacral motor pools is reflected in changes in muscular power production (Dewolf et al., 2019a).

The findings of this study extend the available information on age-related differences in the neuromuscular control of gait occurring when moving upwards or downwards. Our results further corroborate the idea that aging affects principally the distal (sacral) segments. In addition, we observed that other age-related changes occurred in tasks requiring greater ankle push-off, such as walking uphill. The knowledge gained in the present study may be relevant for the design of preventive or rehabilitative approaches to address the decline of gait performance and mobility in older adults.

## Data Availability Statement

The raw data supporting the conclusions of this article will be made available by the authors, without undue reservation.

## Ethics Statement

The studies involving human participants were reviewed and approved by the IRCCS Fondazione Santa Lucia (protocol n CE/PROG749). The patients/participants provided their written informed consent to participate in this study.

## Author Contributions

AD, YI, and FL contributed to the conception and design of the study. AD, FS-L, and GC contributed to data acquisition. AD and DZ organized the database. AD performed the statistical analysis and wrote the first draft of the manuscript. All authors contributed to manuscript revision, read, and approved the submitted version.

## Conflict of Interest

The authors declare that the research was conducted in the absence of any commercial or financial relationships that could be construed as a potential conflict of interest.

## Publisher’s Note

All claims expressed in this article are solely those of the authors and do not necessarily represent those of their affiliated organizations, or those of the publisher, the editors and the reviewers. Any product that may be evaluated in this article, or claim that may be made by its manufacturer, is not guaranteed or endorsed by the publisher.

## Footnotes

1
https://seniam.org

## References

[B1] Aagaard P. Magnusson P. S. Larsson B. Kjaer M. Krustrup P. (2007). Mechanical muscle function, morphology, and fiber type in lifelong trained elderly. *Med. Sci. Sports Exerc.* 39 1989–1996. 10.1249/mss.0b013e31814fb402 17986907

[B2] Anderson D. E. Madigan M. L. (2014). Healthy older adults have insufficient hip range of motion and plantar flexor strength to walk like healthy young adults. *J. Biomech.* 47 1104–1109. 10.1016/j.jbiomech.2013.12.024 24461576 PMC3975833

[B3] Alexander N. Strutzenberger G. Ameshofer L. M. Schwameder H. (2017). Lower limb joint work and joint work contribution during downhill and uphill walking at different inclinations. *J. Biomech.* 61 75–80. 10.1016/j.jbiomech.2017.07.001 28734544

[B4] Arizono N. Koreto O. Iwai Y. Hidaka T. Takeoka O. (1984). Morphometric analysis of human neuromuscular junction in different ages. *Acta Pathol Jpn* 34 1243–1249. 10.1111/j.1440-1827.1984.tb00551.x 6524376

[B5] Berens P. (2009). CircStat: a MATLAB toolbox for circular statistics. *J. Statist. Software* 31 1–21.

[B6] Bianchi L. Angelini D. Orani G. P. Lacquaniti F. (1998). Kinematic coordination in human gait: relation to mechanical energy cost. *J. Neurophysiol.* 79 2155–2170. 10.1152/jn.1998.79.4.2155 9535975

[B7] Bleyenheuft C. Detrembleur C. (2012). Kinematic covariation in pediatric, adult and elderly subjects: is gait control influenced by age? *Clin. Biomechan.* 27 568–572. 10.1016/j.clinbiomech.2012.01.010 22386536

[B8] Borghese N. A. Bianchi L. Lacquaniti F. (1996). Kinematic determinants of human locomotion. *J. Physiol.* 494 863–879. 8865081 10.1113/jphysiol.1996.sp021539 PMC1160684

[B9] Brooks S. V. Faulkner J. A. (1988). Contractile properties of skeletal muscles from young, adult and aged mice. *J. Physiol.* 404 71–82. 10.1113/jphysiol.1988.sp017279 3253447 PMC1190815

[B10] Cappellini G. Ivanenko Y. P. Dominici N. Poppele R. E. Lacquaniti F. (2010). Migration of motor pool activity in the spinal cord reflects body mechanics in human locomotion. *J. Neurophysiol.* 104 3064–3073. 10.1152/jn.00318.2010 20881204

[B11] DeVita P. Hortobagyi T. (2000). Age causes a redistribution of joint torques and powers during gait. *J. Appl. Physiol.* 88 1804–1811. 10797145 10.1152/jappl.2000.88.5.1804

[B12] Dewolf A. H. Ivanenko Y. Zelik K. E. Lacquaniti F. Willems P. A. (2018). Kinematic patterns while walking on a slope at different speeds. *J Appl Physiol.* 125 642–653. 10.1152/japplphysiol.01020.2017 29698109 PMC6842866

[B13] Dewolf A. H. Ivanenko Y. P. Lacquaniti F. Willems P. A. (2017). Pendular energy transduction within the step during human walking on slopes at different speeds. *PLoS One* 12:e0186963. 10.1371/journal.pone.0186963 29073208 PMC5658120

[B14] Dewolf A. H. Ivanenko Y. P. Mesquita R. M. Lacquaniti F. Willems P. A. (2020a). Neuromechanical adjustments when walking with an aiding or hindering horizontal force. *Eur. J. Appl. Physiol.* 120 91–106. 10.1007/s00421-019-04251-4251 31701272

[B15] Dewolf A. H. Mesquita R. M. Willems P. A. (2020b). Intra-limb and muscular coordination during walking on slopes. *Eur. J. Appl. Physiol.* 120 1841–1854. 10.1007/s00421-020-04415-4414 32524225

[B16] Dewolf A. H. Sylos-Labini F. Cappellini G. Lacquaniti F. Ivanenko Y. (2020c). Emergence of different gaits in infancy: relationship between developing neural circuitries and changing biomechanics. *Front. Bioeng. Biotechnol.* 8:473. 10.3389/fbioe.2020.00473 32509753 PMC7248179

[B17] Dewolf A. H. Ivanenko Y. P. Zelik K. E. Lacquaniti F. Willems P. A. (2019a). Differential activation of lumbar and sacral motor pools during walking at different speeds and slopes. *J. Neurophysiol.* 122 872–887. 10.1152/jn.00167.2019 31291150 PMC6734402

[B18] Dewolf A. H. Meurisse G. M. Schepens B. Willems P. A. (2019b). Effect of walking speed on the intersegmental coordination of lower-limb segments in elderly adults. *Gait Posture* 70 156–161. 10.1016/j.gaitpost.2019.03.001 30875602

[B19] Dewolf A. H. Sylos-Labini F. Cappellini G. Ivanenko Y. Lacquaniti F. (2021). Age-related changes in the neuromuscular control of forward and backward locomotion. *PLoS One* 16:e0246372. 10.1371/journal.pone.0246372 33596223 PMC7888655

[B20] Franz J. R. (2016). The age-associated reduction in propulsive power generation in walking. *Exerc. Sport Sci. Rev.* 44 129–136. 10.1249/JES.0000000000000086 27433977 PMC9382873

[B21] Franz J. R. Kram R. (2013). Advanced age affects the individual leg mechanics of level, uphill, and downhill walking. *J. Biomech.* 46 535–540. 10.1016/j.jbiomech.2012.09.032 23122946 PMC3616147

[B22] Franz J. R. Kram R. (2014). Advanced age and the mechanics of uphill walking: a joint-level, inverse dynamic analysis. *Gait Posture* 39 135–140. 10.1016/j.gaitpost.2013.06.012 23850328 PMC3842369

[B23] Gueugnon M. Stapley P. J. Gouteron A. Lecland C. Morisset C. Casillas J.-M. (2019). Age-Related adaptations of lower limb intersegmental coordination during walking. *Front. Bioeng. Biotechnol.* 7:173. 10.3389/fbioe.2019.00173 31380364 PMC6652268

[B24] Hafer J. F. Boyer K. A. (2018). Age related differences in segment coordination and its variability during gait. *Gait Posture* 62 92–98. 10.1016/j.gaitpost.2018.02.021 29544156

[B25] Herssens N. Verbecque E. Hallemans A. Vereeck L. Van Rompaey V. Saeys W. (2018). Do spatiotemporal parameters and gait variability differ across the lifespan of healthy adults? a systematic review. *Gait Posture* 64 181–190. 10.1016/j.gaitpost.2018.06.012 29929161

[B26] Hogan N. (1984). Adaptive control of mechanical impedance by coactivation of antagonist muscles. *IEEE Trans. Automat. Contr.* 29 681–690. 10.1109/TAC.1984.1103644

[B27] Hong S.-W. Leu T.-H. Li J.-D. Wang T.-M. Ho W.-P. Lu T.-W. (2014a). Influence of inclination angles on intra- and inter-limb load-sharing during uphill walking. *Gait Posture* 39 29–34. 10.1016/j.gaitpost.2013.05.023 23800709

[B28] Hong S.-W. Wu C.-H. Lu T.-W. Hu J.-S. Li J.-D. Leu T.-H. (2014b). Biomechanical strategies and the loads in the lower limbs during downhill walking with different inclination angles. *Biomed. Eng. Appl. Basis Commun.* 26:1450071. 10.4015/S1016237214500719

[B29] Hortobágyi T. DeVita P. (2000). Muscle pre- and coactivity during downward stepping are associated with leg stiffness in aging. *J. Electromyogr. Kinesiol.* 10 117–126. 10.1016/s1050-6411(99)00026-27 10699559

[B30] Hortobágyi T. Rider P. Gruber A. H. DeVita P. (2016). Age and muscle strength mediate the age-related biomechanical plasticity of gait. *Eur. J. Appl. Physiol.* 116 805–814. 10.1007/s00421-015-3312-3318 26867788

[B31] Ivanenko Y. P. Dominici N. Cappellini G. Di Paolo A. Giannini C. Poppele R. E. (2013). Changes in the spinal segmental motor output for stepping during development from infant to adult. *J. Neurosci.* 33 3025a–3036a. 23407959 10.1523/JNEUROSCI.2722-12.2013 PMC6619203

[B32] Ivanenko Y. P. Dominici N. Lacquaniti F. (2007). Development of independent walking in toddlers. *Exerc. Sport Sci. Rev.* 35 67–73. 10.1249/JES.0b013e31803eafa8 17417053

[B33] Ivanenko Y. P. Poppele R. E. Lacquaniti F. (2006). Spinal cord maps of spatiotemporal alpha-motoneuron activation in humans walking at different speeds. *J. Neurophysiol.* 95 602–618. 10.1152/jn.00767.2005 16282202

[B34] Janshen L. Santuz A. Ekizos A. Arampatzis A. (2017). Modular control during incline and level walking in humans. *J. Exp. Biol.* 220 807–813. 10.1242/jeb.148957 27980122

[B35] Kendall F. McCreary E. Provance P. Rodgers M. Romani W. (2005). *Muscles. Testing and Function with Posture and Pain.* Baltimore: Lippincott Williams and Wilkins.

[B36] Krupenevich R. L. Clark W. H. Ray S. F. Takahashi K. Z. Kashefsky H. E. Franz J. R. (2021). Effects of age and locomotor demand on foot mechanics during walking. *J. Biomech.* 123:110499. 10.1016/j.jbiomech.2021.110499 34015739 PMC8223147

[B37] Laufer Y. (2005). Effect of age on characteristics of forward and backward gait at preferred and accelerated walking speed. *J. Gerontol. A Biol. Sci. Med. Sci.* 60 627–632. 10.1093/gerona/60.5.627 15972616

[B38] Lay A. N. Hass C. J. Gregor R. J. (2006). The effects of sloped surfaces on locomotion: a kinematic and kinetic analysis. *J. Biomech.* 39 1621–1628. 10.1016/j.jbiomech.2005.05.005 15990102

[B39] Lexell J. (1995). Human aging, muscle mass, and fiber type composition. *J. Gerontol. A Biol. Sci. Med. Sci.* 50 Spec No 11–16. 10.1093/gerona/50a.special_issue.11 7493202

[B40] Ling S. M. Conwit R. A. Ferrucci L. Metter E. J. (2009). Age-Associated changes in motor unit physiology: observations from the baltimore longitudinal study of aging. *Arch. Phys. Med. Rehabil.* 90 1237–1240. 10.1016/j.apmr.2008.09.565 19577038 PMC5496096

[B41] Martino G. Ivanenko Y. Serrao M. Ranavolo A. Draicchio F. Rinaldi M. (2018). Differential changes in the spinal segmental locomotor output in hereditary spastic paraplegia. *Clin. Neurophysiol.* 129 516–525. 10.1016/j.clinph.2017.11.028 29353180

[B42] Martino G. Ivanenko Y. P. d’Avella A. Serrao M. Ranavolo A. Draicchio F. (2015). Neuromuscular adjustments of gait associated with unstable conditions. *J. Neurophysiol.* 114 2867–2882. 10.1152/jn.00029.2015 26378199 PMC4737426

[B43] Martino G. Ivanenko Y. P. Serrao M. Ranavolo A. d’Avella A. Draicchio F. (2014). Locomotor patterns in cerebellar ataxia. *J. Neurophysiol.* 112 2810–2821. 10.1152/jn.00275.2014 25185815

[B44] McFadyen B. J. Winter D. A. (1988). An integrated biomechanical analysis of normal stair ascent and descent. *J. Biomech.* 21 733–744. 10.1016/0021-9290(88)90282-90285 3182877

[B45] McGibbon C. A. (2003). Toward a better understanding of gait changes with age and disablement: neuromuscular adaptation. *Exerc. Sport Sci. Rev.* 31 102–108. 10.1097/00003677-200304000-200304009 12715975

[B46] Meurisse G. M. Bastien G. J. Schepens B. (2019). The step-to-step transition mode: a potential indicator of first-fall risk in elderly adults? *PLoS One* 14:e0220791. 10.1371/journal.pone.0220791 31374108 PMC6677305

[B47] Mian O. S. Thom J. M. Ardigò L. P. Narici M. V. Minetti A. E. (2006). Metabolic cost, mechanical work, and efficiency during walking in young and older men. *Acta Physiol.* 186 127–139. 10.1111/j.1748-1716.2006.01522.x 16497190

[B48] Monaco V. Ghionzoli A. Micera S. (2010). Age-related modifications of muscle synergies and spinal cord activity during locomotion. *J. Neurophysiol.* 104 2092–2102. 20685924 10.1152/jn.00525.2009

[B49] Monaco V. Rinaldi L. A. Macrì G. Micera S. (2009). During walking elders increase efforts at proximal joints and keep low kinetics at the ankle. *Clin. Biomech.* 24 493–498. 10.1016/j.clinbiomech.2009.04.004 19427720

[B50] Montgomery J. R. Grabowski A. M. (2018). The contributions of ankle, knee and hip joint work to individual leg work change during uphill and downhill walking over a range of speeds. *R Soc. Open Sci.* 5:180550. 10.1098/rsos.180550 30225047 PMC6124028

[B51] Noble J. W. Prentice S. D. (2008). Intersegmental coordination while walking up inclined surfaces: age and ramp angle effects. *Exp. Brain Res.* 189 249–255. 10.1007/s00221-008-1464-z 18584161

[B52] Perry M. C. Carville S. F. Smith I. C. H. Rutherford O. M. Newham D. J. (2007). Strength, power output and symmetry of leg muscles: effect of age and history of falling. *Eur. J. Appl. Physiol.* 100 553–561. 10.1007/s00421-006-0247-240 16847676

[B53] Reeves N. D. Spanjaard M. Mohagheghi A. A. Baltzopoulos V. Maganaris C. N. (2009). Older adults employ alternative strategies to operate within their maximum capabilities when ascending stairs. *J. Electromyogr. Kinesiol.* 19 e57–e68. 10.1016/j.jelekin.2007.09.009 18053743

[B54] Rosenberg I. H. (2011). Sarcopenia: origins and clinical relevance. *Clin. Geriatr. Med* 27 337–339. 10.1016/j.cger.2011.03.003 21824550

[B55] Rozumalski A. Steele K. M. Schwartz M. H. (2017). Muscle synergies are similar when typically developing children walk on a treadmill at different speeds and slopes. *J. Biomech.* 64 112–119. 10.1016/j.jbiomech.2017.09.002 28943157

[B56] Saito A. Tomita A. Ando R. Watanabe K. Akima H. (2018). Similarity of muscle synergies extracted from the lower limb including the deep muscles between level and uphill treadmill walking. *Gait Posture* 59 134–139. 10.1016/j.gaitpost.2017.10.007 29031138

[B57] Samson M. M. Crowe A. de Vreede P. L. Dessens J. A. G. Duursma S. A. Verhaar H. J. J. (2001). Differences in gait parameters at a preferred walking speed in healthy subjects due to age, height and body weight. *Aging Clin. Exp. Res.* 13 16–21. 10.1007/BF03351489 11292147

[B58] Santuz A. Brüll L. Ekizos A. Schroll A. Eckardt N. Kibele A. (2020). Neuromotor dynamics of human locomotion in challenging settings. *iScience* 23:100796. 10.1016/j.isci.2019.100796 31962235 PMC6971393

[B59] Santuz A. Ekizos A. Eckardt N. Kibele A. Arampatzis A. (2018). Challenging human locomotion: stability and modular organisation in unsteady conditions. *Sci. Rep.* 8:2740. 10.1038/s41598-018-21018-21014 29426876 PMC5807318

[B60] Seidler R. D. Bernard J. A. Burutolu T. B. Fling B. W. Gordon M. T. Gwin J. T. (2010). Motor control and aging: links to age-related brain structural, functional, and biochemical effects. *Neurosci. Biobehav. Rev.* 34 721–733. 10.1016/j.neubiorev.2009.10.005 19850077 PMC2838968

[B61] Silder A. Heiderscheit B. Thelen D. G. (2008). Active and passive contributions to joint kinetics during walking in older adults. *J. Biomech.* 41 1520–1527. 10.1016/j.jbiomech.2008.02.016 18420214 PMC2702713

[B62] Silverman A. K. Neptune R. R. Sinitski E. H. Wilken J. M. (2014). Whole-body angular momentum during stair ascent and descent. *Gait Posture* 39 1109–1114. 10.1016/j.gaitpost.2014.01.025 24636222

[B63] Skinner H. B. Barrack R. L. Cook S. D. (1984). Age-related decline in proprioception. *Clin. Orthopaed. Related Res.* 184 208–211. 6705349

[B64] Startzell J. K. Owens D. A. Mulfinger L. M. Cavanagh P. R. (2000). Stair negotiation in older people: a review. *J. Am. Geriatr. Soc.* 48 567–580. 10.1111/j.1532-5415.2000.tb05006.x 10811553

[B65] Tomlinson B. E. Irving D. (1977). The numbers of limb motor neurons in the human lumbosacral cord throughout life. *J. Neurol. Sci.* 34 213–219. 925710 10.1016/0022-510x(77)90069-7

[B66] Waanders J. B. Murgia A. Hortobágyi T. DeVita P. Franz J. R. (2020). How age and surface inclination affect joint moment strategies to accelerate and decelerate individual leg joints during walking. *J. Biomech.* 98:109440. 10.1016/j.jbiomech.2019.109440 31690458 PMC7245140

[B67] Williams K. Bird M. (1992). The aging mover: a preliminary report on constraints to action. *Int. J. Aging Hum. Dev.* 34 241–255. 10.2190/93WR-P5N0-34FP-XGMF 1582716

[B68] Winter D. A. Patla A. E. Frank J. S. Walt S. E. (1990). Biomechanical walking pattern changes in the fit and healthy elderly. *Phys. Ther.* 70 340–347. 10.1093/ptj/70.6.340 2345777

